# Flow cytometric analysis identifies changes in S and M phases as novel cell cycle alterations induced by the splicing inhibitor isoginkgetin

**DOI:** 10.1371/journal.pone.0191178

**Published:** 2018-01-16

**Authors:** Erin J. Vanzyl, Kayleigh R. C. Rick, Alex B. Blackmore, Erin M. MacFarlane, Bruce C. McKay

**Affiliations:** 1 Department of Biology, Carleton University, Ottawa ON, Canada; 2 Institute for Biochemistry, Carleton University, Ottawa ON, Canada; Virginia Commonwealth University, UNITED STATES

## Abstract

The spliceosome is a large ribonucleoprotein complex that catalyzes the removal of introns from RNA polymerase II-transcribed RNAs. Spliceosome assembly occurs in a stepwise manner through specific intermediates referred to as pre-spliceosome complexes E, A, B, B* and C. It has been reported that small molecule inhibitors of the spliceosome that target the SF3B1 protein component of complex A lead to the accumulation of cells in the G_1_ and G_2_/M phases of the cell cycle. Here we performed a comprehensive flow cytometry analysis of the effects of isoginkgetin (IGG), a natural compound that interferes with spliceosome assembly at a later step, complex B formation. We found that IGG slowed cell cycle progression in multiple phases of the cell cycle (G_1_, S and G_2_) but not M phase. This pattern was somewhat similar to but distinguishable from changes associated with an SF3B1 inhibitor, pladienolide B (PB). Both drugs led to a significant decrease in nascent DNA synthesis in S phase, indicative of an S phase arrest. However, IGG led to a much more prominent S phase arrest than PB while PB exhibited a more pronounced G_1_ arrest that decreased the proportion of cells in S phase as well. We also found that both drugs led to a comparable decrease in the proportion of cells in M phase. This work indicates that spliceosome inhibitors affect multiple phases of the cell cycle and that some of these effects vary in an agent-specific manner despite the fact that they target splicing at similar stages of spliceosome assembly.

## Introduction

Pre-mRNA splicing is an essential step in the maturation of messenger RNAs (mRNAs) required for the production of proteins in eukaryotic organisms [1]. The removal of introns requires a combination of *cis-*acting elements and *trans-*acting factors. The *cis-*acting elements include the 5’ splice site, the 3’ splice site, the branch point sequence and the polypyrimidine tract. There are five *trans*-acting small nuclear RNAs (snRNAs: U1, U2, U4, U5 and U6) that are associated with many proteins that together form five protein complexes (U1, U2, U4, U5 and U6 snRNPs). Collectively, these snRNPs are required to assemble the major spliceosome that catalyzes 99% of all pre-mRNA splicing [1, 2]. The snRNPs assemble on pre-mRNAs in a sequential manner forming spliceosome complexes E, A, B, B* and C, in succession. The process is initiated when the U1 snRNP binds the 5’ splice site (E complex). Three proteins (SF1, U2AF1 and U2AF2) bind the branch point sequence, the 3' splice site and the polypyrimidine tract of the intron, respectively before the U2 snRNP (including SF3B1) displaces SF1 and binds to the branch point (A complex) [3, 4]. The B complex is then assembled through the addition of the U4, U5 and U6 snRNPs and this complex is activated to permit catalytic activity. Splicing then proceeds through two transesterification reactions ligating exons and releasing the intron [1–3].

There are many small molecule inhibitors of pre-mRNA splicing and many, if not all of them, appear to exhibit anti-neoplastic activity in many cancer cell types [5–9]. Cell cycle analysis based on DNA content indicates that inhibition of pre-mRNA splicing leads to the accumulation of cells with G_1_ and G_2_/M DNA content and a decrease in the proportion of cells in S phase [10–12]. All of the drugs analyzed to date interact with the same protein target, SF3B1 and share a common mechanism of action [7, 10–14]. They interfere with U2 binding to the branch point and thus complex A formation. It was unclear whether these alterations in cell cycle distribution were caused by defects in SF3B1 specifically or if these changes are common to all pre-mRNA splicing inhibitors.

Isoginkgetin (IGG) is a natural compound originally isolated from *Ginkgo biloba* trees that inhibits splicing at a subsequent step in spliceosome assembly [15]. Although the precise target of IGG remains unknown, it prevents the binding of U4/U5/U6 snRNPs to the pre-spliceosome complex, inhibiting B complex formation [15]. Therefore, IGG has a distinct mode of action compared to the SF3B1 inhibitors like pladienolide B (PB). We sought to characterize the effects of IGG on cell cycle distribution using PB for comparison in colon and ovarian cancer cells. We found that IGG slows cell cycle progression in multiple phases of the cell cycle (G_1_, S and G_2_) with a decrease in the M phase population. S phase arrest was the predominant effect detected following IGG treatment while G_1_ arrest was more prominent in PB-treated cells. Therefore, the response of colon cancer cells to IGG was distinguishable from changes associated with the SF3B1 inhibitor PB. This work provides important insight into the relationship between spliceosome inhibitors and cell cycle dysregulation and indicates that some of these cell cycle alterations occur in an agent-specific manner.

## Materials and methods

### Cell culture and drug treatment

HCT116 colon cancer cells, a subline in which p53 was deleted by homologous recombination (p53KO cells) and A2780 ovarian cancer cells were grown in McCoys media (Hyclone) supplemented with 12% serum in a 3 to 1 ratio of newborn calf serum (NBCS) (Gibco) to fetal bovine serum (FBS) (Gibco) plus penicillin and streptomycin antibiotics (Hyclone). Cells were seeded at a density of 5x10^5^ cells per 6 cm dish 24 hours prior to treatment and they were treated in 3 ml of media containing dimethyl sulfoxide (DMSO), IGG or PB. IGG and PB were purchased from EMD Millipore (Etobikoke, ON) while DMSO and colcemid were obtained from Sigma Canada (Oakville, ON).

### Cell cycle analysis

One parameter flow cytometric analysis of cell cycle distribution based on DNA content was performed as previously described [16]. Briefly, cells were collected by following trypsin treatment. Cells were rinsed twice with phosphate buffered saline (PBS pH 7.4) and collected by centrifugation. Pellets were resuspended in ice cold 70% ethanol and stored at -20°C for a minimum of 30 min. Cells were collected by centrifugation, rinsed twice in PBS and resuspended in 20 μg/ml propidium iodide (PI) in PBS with 10–50 μg/ml RNase A [16]. Cells were stored at 4°C for a minimum of 30 min. Fluorescence (FL2) was measured using a BD Accuri C6 flow cytometer and cell cycle phase was estimated from histograms using Modfit 4.1 software (Verity Software House, Topsham, ME).

Two parameter flow cytometric analysis was performed as previously described [17, 18]. Briefly, one hour prior to collection, the medium was replaced with fresh medium containing 30 uM 5’Bromo-2’deoxyuridine (BrdU) (Sigma). Cells were washed with PBS, detached with trypsin and collected by centrifugation with two washes in PBS. Cells were then fixed in 70% ethanol for a minimum of 1 hour at -20°C. Fixed cells were washed in PBS and collected by centrifugation before being resuspended in PBS with 50 μg/ml RNAse A and incubated for 30 minutes at 37°C. Samples were centrifuged and the cells were resuspended in 0.1M HCl 0.7% Triton X-100 and incubated on ice for 15 minutes. Cells were collected again, resuspended in sterile H_2_0, boiled for 15 minutes and immediately placed on ice for 15 minutes. One ml of 0.5% Tween 20 in PBS was added to the cell solution and cells were spun down. 0.5 ml of an Alexa Fluor 488-conjugated anti-BrdU antibody (BD Biosciences) in PBS, 5% FBS and 0.5% Tween 20 was added and incubated for 30 minutes. Cells were spun down and then resuspended in 30 μM propidium Iodide (PI) with RNase A. Samples were stored at 4°C for a minimum of 30 min prior to analysis of BrdU incorporation (FL1) and DNA content (FL2) using a BD Accuri C6 benchtop flow cytometer and BD Accuri C6 software.

### Detection of phosphorylated histone H3

Cell monolayers were rinsed with PBS and cells were collected with trypsin, as described above. The cell pellets were resuspended in 4% formaldehyde and incubated for 10 minutes at 37°C. The cells were then chilled on ice for 1 minute, permeabilized in 90% ice cold methanol, and incubated on ice for 30 minutes. Cells were counted with a TC20 Automated Cell Counter (BioRad) and 1x10^6^ cells from each sample were stained. Cells were rinsed in 2–3 ml of incubation buffer (0.5% BSA in PBS) and collected by centrifugation. Cells were resuspended in 100 μl of Alexa Fluor 488-conjugated anti-phospho Histone H3 (Ser10) Antibody (EMD Millipore cat#:06-570-AF488) diluted 1:50 in incubation buffer for 1 hour. Cells were washed in 2 ml incubation buffer and collected by centrifugation. Cells were resuspended in 200 μl of 30 μM PI stain for 30 minutes. Analysis of histone H3 phosphorylation (FL1) and DNA content (FL2) was performed using a BD Accuri C6 benchtop flow cytometer and BD Accuri C6 software.

### Immunoblotting

Cell monolayers were rinsed with PBS and collected in 300 ul RIPA Buffer (Sigma). Samples were sonicated and the protein was quantified using the Bio-Rad Protein Assay (Bio-Rad). Equal amounts of protein were denatured in NuPAGE™ LDS Sample Buffer (Fisher Scientific) with dithiothreitol (DTT), heated at 70°C, loaded onto NuPAGE 4–12% Bis-Tris gels (Fisher Scientific) and transferred to a nitrocellulose membrane (Bio-Rad). Transferred proteins were visualized using 1 mg/ml Ponceau S Red in 1% glacial acetic acid. Membranes were blocked in 5% Milk in Tris Buffered Saline Tween (TBST) pH 8.0 (Tris Buffered Saline, 0.1% Tween 20) for 1 hour. Membranes were incubated overnight at 4°C in primary antibody in 0.5% Milk in TBST pH 8.0. Membranes were washed 4x5 min in TBST and left in secondary antibody (HRP-conjugated goat anti-mouse or goat anti-rabbit from Santa Cruz) for 1–3 hours. Membranes were washed 4x5 minutes in TBST then incubated for 5 minutes in 1 ml of Super Signal West Pico Chemiluminescent Substrate (Thermo Fisher Scientific) and imaged with a Fusion FX5 gel documentation system (Vilber Lourmat). Membranes were stripped using Restore PLUS Western Blot Stripping Buffer (Fisher Scientific) and probed sequentially with several antibodies. Primary antibodies included beta-actin (A2228, Sigma-Aldrich), Cyclin B (554176, BD Pharmingen), Cyclin E (sc-198, Santa Cruz), and Cyclin A (Sc-751, Santa Cruz).

## Results

### Isoginkgetin treatment led to multiple changes in cell cycle progression

**Fig 1:**
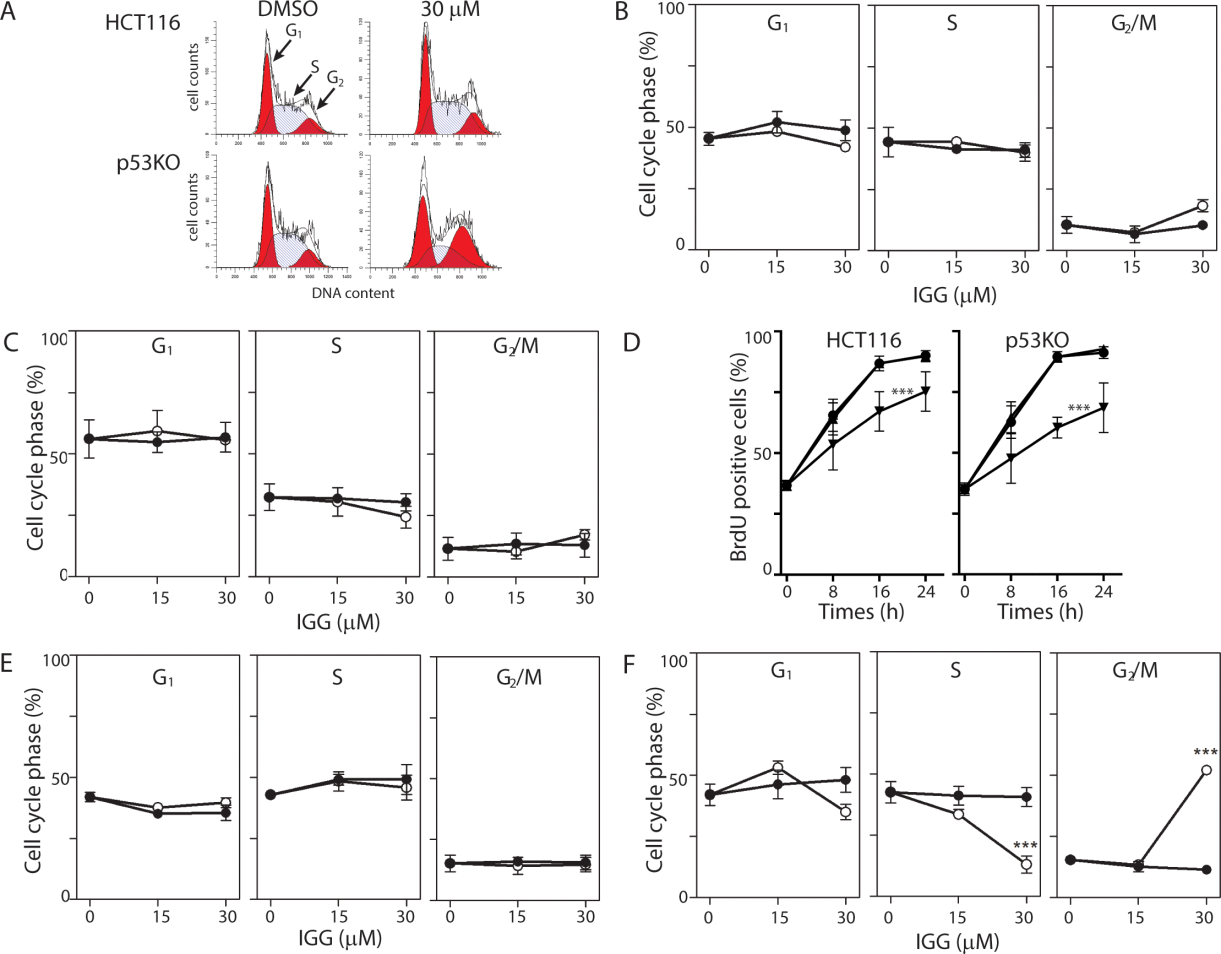
Similar effects on cell growth but differences in cell cycle distribution in IGG treated HCT116 and p53KO cells. HCT116 and p53KO cells were incubated in 15 μM IGG, 30 μM IGG or vehicle control for either 8 or 24 hours. One-parameter flow cytometric analysis of PI stained cells was used to determine cell cycle distributions (G_1_, S and G_2_/M) based on DNA content, using Modfit 4.1 cell cycle analysis software. (A) Representative histograms are presented for samples collected 24 hours following exposure to 30 μM IGG and its vehicle control. (B and C) The compiled cell cycle results from similar analysis of HCT116 cells collected at 8 (B) and 24 (C) hours following treatment with the indicated concentration of IGG (open symbols) and the corresponding vehicle controls (closed symbols). (D) HCT116 and p53KO (left and right panels, respectively) were incubated in growth medium alone (circles), DMSO (triangles) or IGG (inverted triangles) along with BrdU for the indicated period. The proportion of cells incorporating BrdU at each time point was estimated by flow cytometry. (E and F) p53KO cells were incubated in IGG for either 8 (D) or 24 hours (E) at the indicated concentration (open symbols) or with an equivalent volume of DMSO (closed symbols). Each value in B through F represents the mean (+/- SEM) determined from a minimum of 3 independent experiments. *** indicates that the value is significant different (P<0.001) from controls (DMSO and no drug) by one way ANOVA followed by Tukey multiple comparisons test.

HCT116 colon cancer cells were exposed to various concentrations of IGG, up to the reported IC50 (30 μM)[15] and cells were collected at either 8 or 24 hours for flow cytometric analysis based on DNA content. The proportion of cells in the G_1_, S and G_2_/M phases of the cell cycle was estimated using Modfit 4.1 software (Fig 1A). No significant alteration of cell cycle distribution at either time point was detected (Fig 1B and 1C). This finding was perplexing given previous analysis of splicing inhibitors [10–12, 19]. Therefore, we measured BrdU incorporation in IGG-treated HCT116 cells. Fewer IGG-treated cells incorporated BrdU compared to untreated and DMSO-treated controls (Fig 1D). These results imply that the cytostatic effects exerted by IGG were exerted roughly equally across all phases of the cell cycle.

We considered that the G_1_ and G_2_/M arrests reported in response to SF3B1 inhibitors were detected using WiDr colon cancer cells and Chinese hamster ovary (CHO) cells, respectively [10–12]. Both of these cell lines express mutant p53 [20, 21], an important cell cycle regulatory protein, while HCT116 cells express wildtype p53 [22]. Therefore, we considered that p53-deficient cells could respond differently to IGG treatment. IGG led to a similar decrease in BrdU incorporation in an isogenic HCT116-derived p53 knockout (p53KO) cell line (Fig 1D) [22]. One parameter flow cytometric analysis based on DNA content indicated that there were indeed differences in cell cycle alterations in the isogenic pairs of cell lines (Fig 1E and 1F). Here we detected a significant increase in the proportion of cells with 4C DNA content (G_2_ or M) with a corresponding decrease in the proportion of cells in S phase (Fig 1E). The decrease in the proportion of cells in S phase was consistent with a p53-independent G_1_ arrest while the sizeable accumulation of cells in G_2_ or M is indicative of an arrest in the G_2_ and/or M phases of the cell cycle. G_2_ and M phases cannot be distinguished by DNA content alone. Importantly, the changes observed in p53KO cells more closely paralleled those reported previously in other p53-deficient cell lines [10–12].

### IGG led to a decrease in the proportion of M phase cells

**Fig 2:**
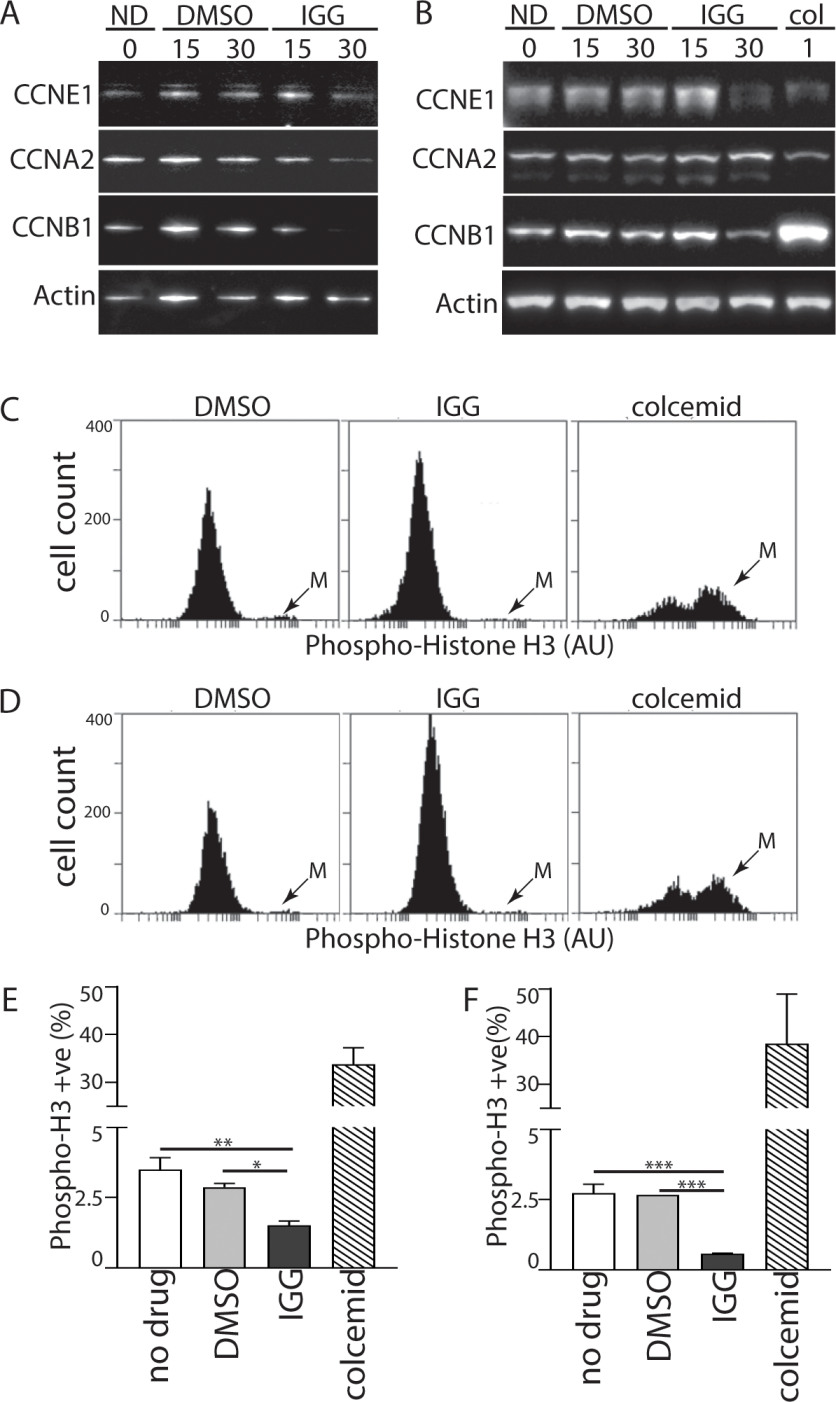
Markers of M phase are decreased in response to IGG. (A) HCT116 cells were incubated in IGG for 24 hours. Protein lysates were collected and analyzed by immunoblot using antibodies raised against the indicated proteins. (B) p53 KO cells were treated as described in (A) except that colcemid, a microtubule-depolymerizing agent, was included in lane 6. HCT116 (C) and p53KO (D) cells were exposed to DMSO, IGG or colcemid for 24 hours and the M phase-specific phosphorylation of histone H3 was detected using a phosphospecific antibody coupled with flow cytometry. ‘M’ denotes the mitotic population. The proportion of phospho-H3 positive cells was determined from 3 independent experiments (E and F). *, ** and *** denote that the values were significantly different (P 0.05, 0.01, 0.001, respectively) by one way ANOVA followed by a Tukey multiple comparisons test. Statistical analysis of colcemid, the positive control, was not included for clarity.

To distinguish between G_2_ and M phase arrest, we used two different methods. First, we examined the expression of cyclins E1, A2 and B1 because cyclin E1 is expressed through the G_1_ to S phase transition, cyclin A2 is expressed in S phase and G_2_ phases predominantly while cyclin B1 is the predominant mitotic cyclin (see [23] for review). In HCT116, there was little change in cyclin E1 or cyclin A2 expression (Fig 2A). This correlated well with measured cell cycle distributions in G_1_, S and G_2_ phases, supporting the fact that cell cycle distribution is unaltered in HCT116 cells in response to IGG treatment. Therefore, the cytostatic effects of IGG on HCT116 proliferation (Fig 1C) are likely associated with a slowdown across multiple phases of the cell cycle. The decrease in cyclin B1 suggested that the proportion of cells in M phase may have decreased, however this population is usually small and would not affect the apparent cell cycle distribution greatly.

In p53KO cells that had exhibited a loss of S phase and an increase in G_2_/M following IGG treatment (recall Fig 1E); we detected a decrease in the expression of cyclins E1 and B1 with only very minor changes in cyclin A2 expression (Fig 2B). Decreased expression of cyclin E1 suggests that fewer cells were present in late G_1_ and early S phase. The expression of cyclin A2 remained unchanged and this was consistent with offsetting effects on S and G_2_ phases because both of these populations of cells would be cyclin A2 positive. Once again, cyclin B1 expression decreased in response to IGG while our positive control, the microtubule-depolymerizing drug colcemid increased cyclin B1 expression, as expected (Fig 2B)[24]. Therefore, the large accumulation of p53 KO cells with 4C DNA content (recall Fig 1E) is best accounted for by G_2_ accumulation and not M phase arrest.

To test this assertion further, we used flow cytometry to detect the proportion of cells carrying the mitosis-specific phosphorylation of histone H3 on serine 10 (phospho-H3) [25]. The proportion of phospho-H3 positive HCT116 and p53KO cells represented only a small fraction of the total population in control samples and this small fraction decreased further in the presence of IGG (Fig 2C and 2D). In contrast to IGG, the M phase arrest associated with colcemid treatment led to a remarkable increase in the proportion of phospho-H3 positive HCT116 and p53KO cells (Fig 2C and 2D). Quantitative analysis of histograms derived from multiple independent experiments indicated that the threefold decrease in phospho-H3 positive cells was statistically significant (Fig 2E and 2F). Therefore, the accumulation of p53KO cells with 4C DNA content did not result from a mitotic arrest but rather it is consistent with a G_2_ cell cycle arrest.

### IGG led to a pronounced reversible S phase arrest

**Fig 3:**
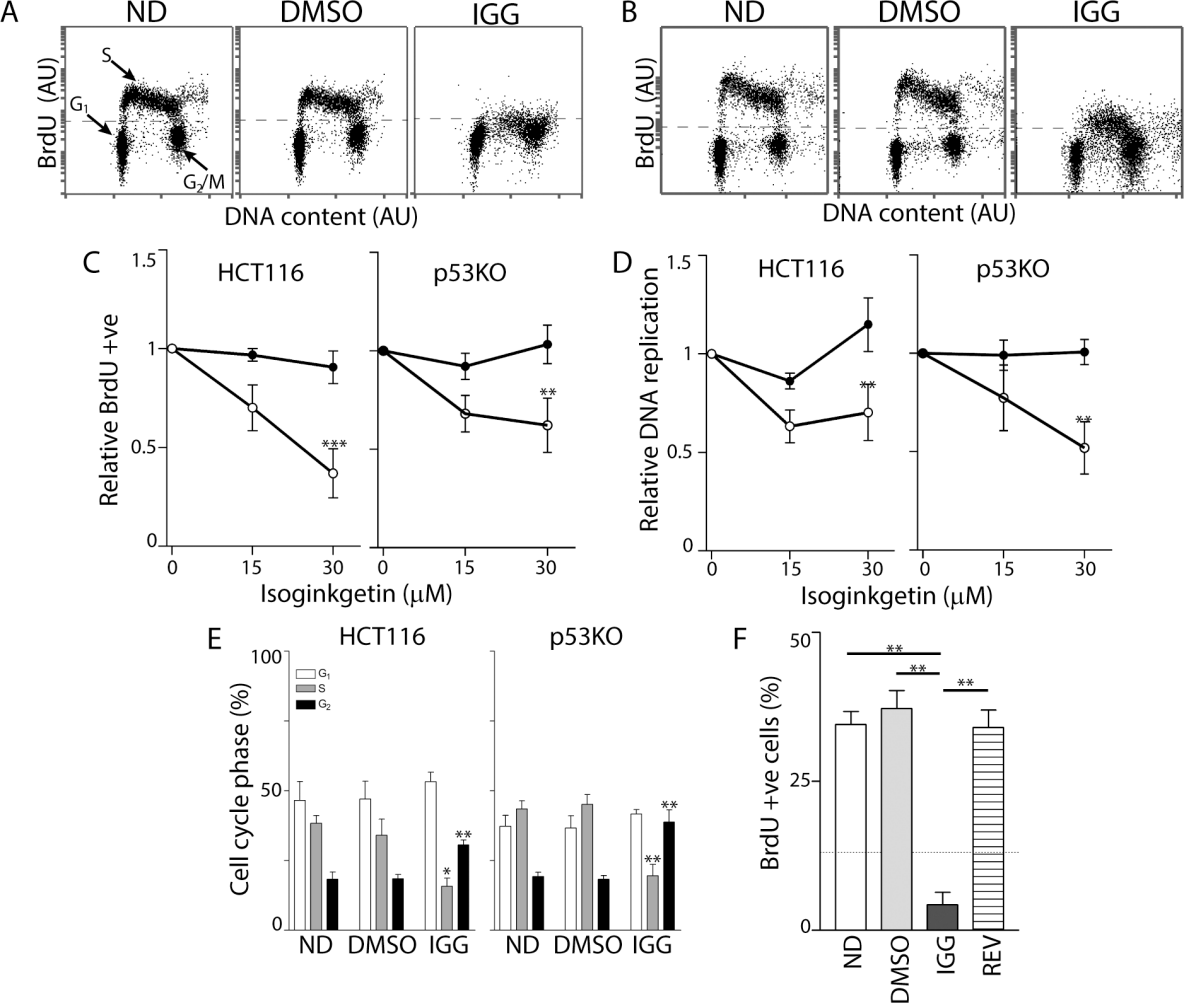
IGG induces S phase arrest in HCT116 and p53KO cells. Two-parameter flow cytometry analysis of BrdU incorporation and DNA content was performed following a 24-hour exposure of HCT116 (A) and p53 KO (B) cells to 30 μM IGG. (C) The proportion of BrdU positive cells was determined and expressed relative to untreated controls. (D) Relative replication is an estimate of the efficiency of DNA replication in the BrdU positive population of cells. (E) Cell cycle distribution of no drug- (ND), vehicle control- (DMSO) and 30 IGG-treated cells was estimated from dot plots like those presented in A and B. (F) HCT116 cells were exposed to either DMSO for 48 hours (DMSO) or IGG for 24 hours (dashed line) followed by either an additional 24 hours in IGG or fresh medium with DMSO (REV). Data obtained from multiple experiments were expressed as the mean percentage of BrdU positive cells. Each value in C-F represents the mean (+/- SEM) determined from 4, 4, 6 and 3 independent experiments, respectively. In C and D, ** indicates that the values were significantly different (P < 0.01) by two way ANOVA followed by Bonferroni post hoc tests. In E and F, * and ** denote that the value was significantly different (P < 0.05 or P< 0.01) by one-way ANOVA followed by Tukey multiple comparisons test.

We found no change in cell cycle distribution based on DNA content alone in IGG-treated HCT116 cells despite inhibition of DNA replication (recall Fig 1A–1C). We performed two-parameter flow cytometry analysis of BrdU incorporation and DNA content to better separate early and late S phase cells from those cells with similar DNA content (G_1_ and G_2_, respectively). Exposure of HCT116 cells to 30 μM IGG for 24 hours almost completely inhibited BrdU incorporation (Fig 3A). This effect was slightly less pronounced in the p53KO cells (Fig 3B). Nonetheless, the proportion of IGG-treated cells incorporating BrdU decreased significantly in both cell lines, so that there was no longer a clear distinction between S and G_2_ phases (Fig 3C). The proportion of cells with S phase DNA content that incorporated BrdU decreased significantly in the presence of IGG (cells above dashed line in Fig 3A and 3B). Even among cells that had incorporated BrdU, there was a significant decrease in BrdU incorporation per cell (Fig 3D). Quantitation of cell cycle distribution using this more precise method indicated that there was an overall decrease in the proportion of cells in S phase with a corresponding increase in the G_2_ phase of the cell cycle in both cell lines (Fig 3E). However, the proportion of cells in G_2_ is likely overestimated in Figs 1 and 3 because cells arrested in late S phase cannot be distinguished from G_2_ and IGG clouded the S/G_2_ boundary. Overall, the dominant effect detected both qualitatively (Fig 3A and 3B) and quantitatively (Fig 3C and 3D) was the remarkable decrease in DNA synthesis in the presence of IGG (Fig 3A and 3B). This S phase arrest was reversible because removal of IGG permitted HCT116 cells to resume DNA synthesis (Fig 3F). Taken together, IGG induced a pronounced S phase arrest in both cell lines and this arrest was reversible.

**Fig 4:**
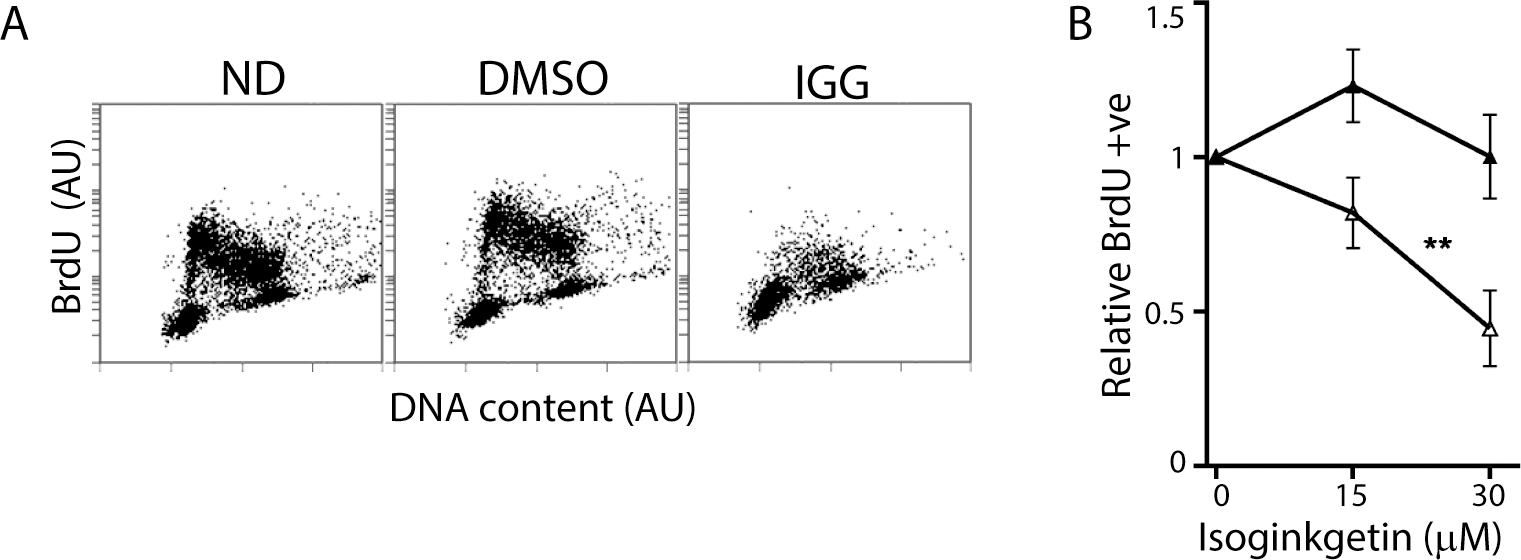
IGG induces S Phase arrest in A2780 ovarian cancer cells. (A) Two-parameter flow cytometric analysis of BrdU incorporation and DNA content was performed following a 24-hour exposure of A2780 cells to IGG. (B) The proportion of vehicle control (closed symbols) and IGG-treated (open symbols) BrdU positive cells was determined. These values are expressed relative to untreated controls. Each value in B represents the mean (+/- SEM) determined from 3 independent experiments. ** denotes that the value was significantly different (P < 0.01) by two way ANOVA followed by Bonferroni post hoc tests.

These HCT116-derived cell lines are isogenic so it was possible that these cells were unique in their S phase response to IGG exposure. Therefore, we also examined the response of A2780 ovarian cancer cells to IGG treatment. Consistent with the results in HCT116 and the p53KO cell lines, replication decreased in A2780 cells treated with 30 μM IGG (Fig 4A). The proportion of BrdU positive S phase cells decreased significantly (Fig 4B). Therefore, IGG induced an S phase arrest in cell lines with distinct origin.

### PB treatment leads to decreased replication and H3 phosphorylation

**Fig 5:**
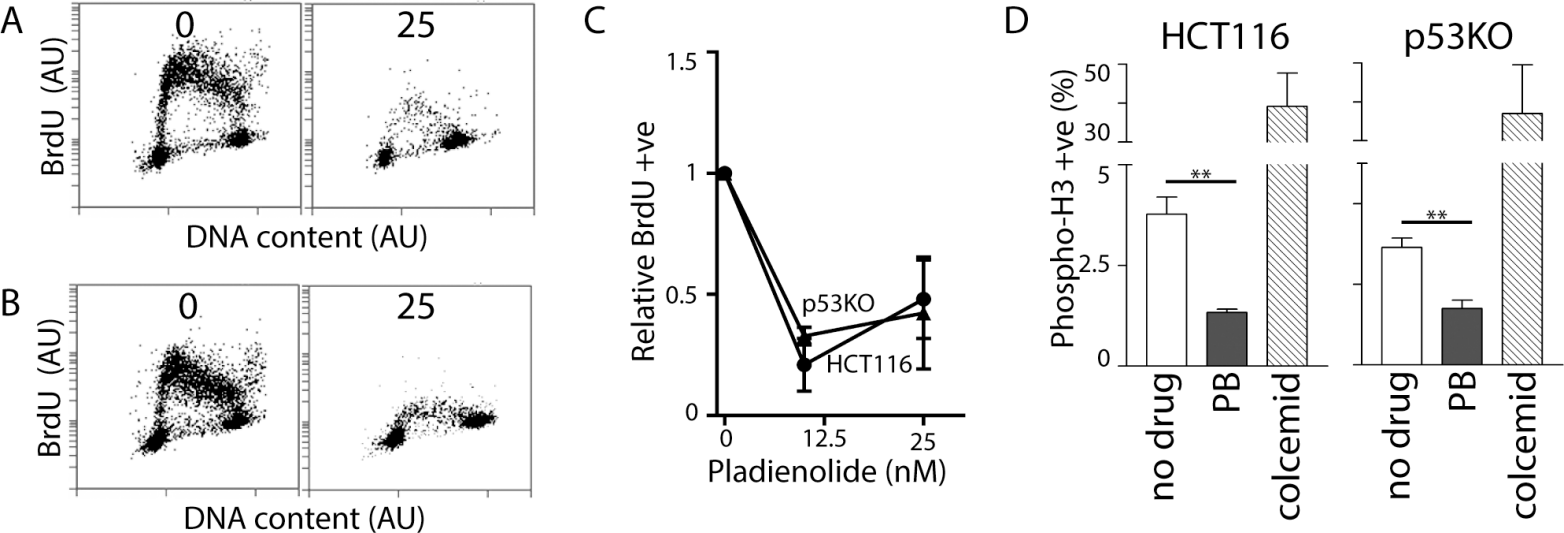
Effects of PB on S and M phases of the cell cycle. HCT116 and p53KO cells were exposed to either 0 or 25 nM PB for 24 hours. HCT116 (A) and p53KO cells (B) were BrdU labelled for 1 hour prior to collection. BrdU incorporation was then assessed by two-parameter flow cytometric analysis. (C) The proportion of cells incorporating BrdU was determined and this is expressed relative to untreated control cells. (D) Phospho-H3 was detected as described in Fig 2. Each value in (C) and (D) represents the mean (+/- SEM) determined from 3 independent experiments. In (C), the mean values for both cell lines at 10 and 25 nM PB were significantly different from 1 by single sample T test (P < 0.05). In (D), ** indicates that the values were significantly different (P<0.01) by Student T test.

The predominant cell cycle alterations reported previously in response to spliceosome inhibition are the accumulation of cells with G_1_ and G_2_ DNA content with a decrease in the proportion of cells with S phase DNA content [10–12]. However, previous cell cycle analysis was limited to one-parameter flow cytometric analysis based on DNA content. Here we performed two-parameter flow cytometric analysis of BrdU labelled cells to determine if a similar S phase was induced by PB. Two prominent changes in S phase were noted in both HCT116 and p53KO cells. There was a large decrease in the proportion of BrdU positive cells, as observed in response to IGG (Fig 5A–5C). However unlike IGG, the total number of cells with S phase DNA content also decreased remarkably (Fig 5A and 5B). Therefore, the decrease in BrdU positive cells wasn’t determined exclusively by decreased DNA replication in S phase cells. Instead, decreased BrdU incorporation resulted from a decrease in the proportion of cells in S phase. This is indicative of a prominent G_1_ arrest following PB but not IGG treatment. Nonetheless, the small number of cells with S phase DNA content had incorporated less BrdU per cell in the presence of PB, consistent with reduced replication in those cells that had escaped the G_1_ arrest and entered S phase (Fig 5A and 5B). Therefore, the relative contribution of G_1_ and S phase arrests to the overall cell cycle distribution was different in response to each of these drugs.

## Discussion

Pre-mRNA splicing is an essential step required to produce mature mRNAs so it is not entirely surprising that perturbations of this processing step would have important consequences on cell biology. Here we found that IGG treatment arrested cell cycle progression at several phases of the cell cycle (G_1_, S and G_2_) in colon and ovarian cancer cell lines, although the relative contribution of each is different among cell lines and drugs tested. Collectively, our results support a model in which disruption of pre-mRNA splicing is linked to cell cycle regulation in multiple phases of the cell cycle. The present work extends previous evidence linking pre-mRNA splicing defects to alterations in mitotic cell division.

The earliest evidence linking pre-mRNA splicing defects with cell cycle alterations stem from early studies in yeast. Temperature sensitive cell division cycle mutants (cdc mutants) isolated in *Saccharomyces cerevisiae* were instrumental in identifying proteins required for progression through the cell cycle [26, 27]. Strains carrying temperature sensitive mutations in the CDC40/PRP17 gene accumulate intermediates of the splicing reaction and undergo G_1_/S and mitotic arrest at the restrictive temperature [28–30]. Furthermore, 8 other splicing factors are synthetic lethal with CDC40/PRP17: PRP8, PRP16, PRP22, SLU7, SLT11, SNR20, CLF1/SYF3, SYF1 and SYF2 [30]. Like CDC40/PRP17, depletion of these proteins also yielded a variety of mitotic defects [30] so mutations in a variety of pre-mRNA splicing factors inhibit cell cycle progression.

There is also more recent genetic evidence to support a similar link between pre-mRNA splicing and cell cycle progression in higher eukaryotes. In *Drosophila melanogaster* S2 cells, 142 genes were identified in a large scale screen for mitotic regulators and 13 of these were splicing factors that, when depleted, gave rise to a defect in sister chromatid separation [31]. Notably, homologues of *S*. *cerevisiae* PRP17, PRP8 and SYF1 were among the splicing factors identified [31]. Similarly, Kittler and coworkers screened a library of 5305 endoribonuclease-prepared short interfering RNAs to identify genes required for cell cycle progression in HeLa cells [32]. Thirty-seven genes were identified that were essential for cell division and 7 were splicing factors. Once again depletion of these splicing factors gave rise to mitotic arrest with abnormal spindle formation [32]. Collectively, genetic evidence links pre-mRNA splicing to cell cycle progression, primarily through M phase defects.

Small molecule inhibitors of the spliceosome did not phenocopy mitotic defects in these models. Inhibitors of SF3B1 reportedly induce the accumulation of cells in G_1_ and G_2_/M phases with a depletion of the S phase population [10, 11, 19, 33]. Our observed G_1_ and G_2_ arrests following PB treatment were generally consistent with these earlier reports except that we did not detect M phase arrest. Previous methodology did not allow assessment of M phase. We also found that IGG led to a slowdown in all phases of the cell cycle except M phase. Thus, cell cycle alterations associated with the small molecule inhibitors did not resemble the results obtained following RNA interference or in genetic models of spliceosome dysfunction. In fact, the two inhibitors assessed in the present work did not lead to identical changes in cell cycle kinetics.

Perhaps the most striking cell cycle phenotype identified here was the striking decrease in DNA replication observed in response to IGG and PB. The overall decrease was the result of the combined effects of G_1_ and S phase arrests. G_1_ arrest delays or prevents entry in to S phase, inhibiting DNA synthesis and S phase arrest leads to decreased nascent DNA synthesis in S phase. PB led primarily to a decrease in the proportion of cells in S phase due to G_1_ arrest. Yet a small fraction of PB-treated cells had S phase DNA content but incorporated BrdU poorly. In contrast, there was no decrease in the proportion of IGG-treated HCT116 cells in S phase. Therefore, the inhibition of nascent DNA synthesis in these IGG-treated cells resulted in large part from a pronounced S phase arrest.

Madrasin is a recently described splicing inhibitor that interferes with the transition from complex A to B [19]. One-parameter flow cytometric analysis of the cellular response to madrasin suggested that this drug leads to the accumulation of cells in S, G_2_ and M phase [19]. These authors did not perform two-flow cytometric analysis to determine if the accumulation of cells in S phase was associated replication arrest. Nonetheless, they noted that nascent DNA synthesis was inhibited using fluorescence microscopy of 5-ethynyl-2′-deoxyuridine (EdU) incorporation [19]. However, the inhibition of DNA synthesis did not correlate well with the fraction of cells in S phase so it isn’t clear if madrasin decreased DNA synthesis through G_1_ and/or S. Nonetheless, our work coupled with this previous publication suggests spliceosome inhibitors prevent normal DNA replication.

Intriguingly, the only stage of the cell cycle for which we found no evidence of an arrest was M phase. Instead, we found that both IGG and PB led to decreased phosphorylation of histone H3 on serine 10. This specific phosphorylation is indicative of M phase [25]. Our positive control colcemid, a microtubule-depolymerizing drug that induces M phase arrest [24], led to a pronounced accumulation of cells expressing phosphorylated H3 protein, as expected. The decrease in M phase was simultaneously associated with decreased expression of the M phase cyclin B1. Together, our results failed to support the existence of an M phase arrest following chemical inhibition of pre-mRNA splicing and clearly distinguish the cellular responses to spliceosome inhibitors from genetic models of spliceosome dysfunction in *S*. *cerevisiae*, *D*. *melanogaster* and HeLa cells in which spindle defects and M phase arrest were the predominant cell cycle related alterations [28–32].

Lastly, we detected subtle differences in the response of isogenic cell lines differing in p53 status. The p53KO cell line appeared to accumulate in G_2_ concomitant with a decrease in the proportion of cells in S phase following IGG treatment (recall Fig 1E). However, the more informative two-parameter flow cytometric method suggested that this increase in G_2_ in IGG-treated p53KO cells was less pronounced than seen when analyzing DNA content alone. It is likely that cell cycle analysis based on DNA content alone overestimated the G_2_ accumulation because G_2_ phase was difficult to distinguish from late S phase in both cell lines due to the arrest of cells in late S phase (recall Fig 3A and 3B). None of the changes in cell cycle distribution observed in response to IGG or PB fit the simple model of p53-dependent cell cycle arrest. The p53 protein is a well-known tumour suppressor that functions as a sequence-specific transcription factor [34]. The cyclin-dependent kinase inhibitor p21^WAF1^ is transcriptionally regulated by p53 and increased p21^WAF1^ leads to G_1_ and G_2_ arrests in response to p53 activation [22, 35, 36]. Here the IGG-induced G_2_ arrest may have been somewhat larger in the absence of p53 but it displayed a similar S phase arrest and equivalent alterations in M phase.

At present, there is no mechanism that links splicing defects to cell cycle control. It may be that pre-mRNA splicing inhibitors lead to decreased production of rate-limiting proteins required for cell cycle progression. Alternatively, there may be specific splicing checkpoints that detect the integrity of this mRNA processing step and respond appropriately. Future efforts will be directed at determining how inhibition of spliceosome function leads to cell cycle arrest in these various phases of the cell cycle, how p53 affects cell cycle alterations and why these small molecule inhibitors of the spliceosome appear to interfere with the cell cycle differently than RNA interference strategies.
