# Correction: Flow cytometric analysis identifies changes in S and M phases as novel cell cycle alterations induced by the splicing inhibitor isoginkgetin

**DOI:** 10.1371/journal.pone.0320500

**Published:** 2025-03-17

**Authors:** Erin van Zyl, Kayleigh R. C. Rick, Alex B. Blackmore, Erin M. MacFarlane, Bruce C. McKay

The first author’s name is spelled incorrect. The correct name is: Erin van Zyl. The correct citation is: van Zyl E, Rick KRC, Blackmore AB, MacFarlane EM, McKay BC (2018) Flow cytometric analysis identifies changes in S and M phases as novel cell cycle alterations induced by the splicing inhibitor isoginkgetin. PLoS ONE 13(1): e0191178. https://doi.org/10.1371/journal.pone.0191178z
